# Effect of bulbospongiosus muscle injection with botulinum-A toxin for treatment of lifelong premature ejaculation; a randomized controlled trial

**DOI:** 10.1038/s41443-024-00831-8

**Published:** 2024-02-02

**Authors:** Khaled Almekaty, Ahmed Ghaith, Maged Ragab, Ayman Rashed, Ayman Hagras, Ayman Ghoneem, Amr Abdel Raheem, Mohamed H. Zahran

**Affiliations:** 1 Urology Department, Tanta University, Tanta, Egypt; 2 Urology Department, 6th of October University, Cairo, Egypt; 3 Andrology Department, Cairo University, Cairo, Egypt; 4 Mansoura Urology and Nephrology Centre, Mansoura, Egypt

**Keywords:** Drug therapy, Medical research

## Abstract

This study aimed at assessing a new line of treatment for lifelong premature ejaculation which is botulinum-A toxin injection into the bulbospongiosus muscle. Sixty patients with lifelong premature ejaculation were independently randomized into 2 groups; group I, 100 U botulinum-A toxin at 10 U/ml saline was injected with ultrasound guidance into the bulbospongiosus muscle and group II which was injected with similar volume of saline. The primary outcome was to compare both groups for changes in the Premature Ejaculation Profile (PEP), Intravaginal Ejaculatory Latency Time (IELT) and partner’s satisfaction at 1, 3 and 6 months after intervention. The second outcome was to compare the adverse events in both groups. Fifty-seven patients completed the study. In group I, the mean PEP increased significantly at 1- (*P* = 0.02) and 3- months (*P* = 0.04) with insignificant increase at 6-month (*P* = 0.6) of follow-up. Also, no significant changes had been noted in IELT or partner’s satisfaction scores throughout the study duration (*P* > 0.05). In group II, no significant changes had been noted in the PEP, IELT and partner’s satisfaction scores throughout the study duration (*P* > 0.05). There were insignificant differences in the changes in the mean PEP (*P* = 0.7, 0.6 and 0.4), IELT (*P* = 0.6,0.6 and 0.5) and partner’s satisfaction scores (*P* = 0.5,0.7 and 0.3) in comparison to the baseline values at 1-, 3- and 6- months, respectively between both groups. Adverse events were observed in only 3 patients (5.3%). In group I, mild erectile dysfunction and post micturition dribbling were reported in one patient each. Where in group II, one patient reported bleeding per urethra (*P* = 0.5). To conclude, injection of botulinum-A toxin into bulbospongiosus seems to be safe but failed to prove clinical efficacy for treatment of lifelong premature ejaculation when compared to placebo.

## Introduction

The complaint of ejaculating prematurely is considered one of the most common forms of sexual dysfunction affecting men whereas its lifelong premature ejaculation (PE) subtype is relatively rare (with an estimated prevalence of 2–5%) [1, 2]. Many definitions have been proposed for lifelong PE, the most accepted one is that of the International Society of Sexual Medicine which consider PE “ejaculation which always or nearly always occurs prior to or within one minute of vaginal penetration from first sexual experiences plus negative personal consequences such as distress, bother, frustration, and/or the avoidance of sexual intimacy” [3]. However, there is no doubt that lifelong PE negatively affects the quality of life of the patient and his partner [4]. Proper assessment of this problem in an objective validated way has always been a challenge.

Treatment of PE varied from behavioral techniques, selective serotonin re-uptake inhibitors and local anesthetics with reported variable outcomes, unsatisfactory for many patients [4]. New lines have been always evolving trying to address this resistant category of patients such as injection of the glans penis with filler [5] and neurectomy of the dorsal nerve of the penis [6].

Injection of bulbospongiosus muscle (BS) with botulinum A-toxin theoretically can inhibit its stereotyped rhythmic contractions during the reflex of ejaculation [7]. Herein, we aimed at assessing the role of botulinum A-toxin injection into BS for treatment of PE in a prospective, randomized, placebo-controlled study.

## Patients and Methods

### Study design

A single-center prospective randomized study was conducted at the Andrology Unit of Tanta University in Egypt between November 2020 and November 2022 to evaluate the efficacy and safety of botulinum-A toxin injection into BS in the treatment of lifelong PE in a sample of Egyptian patients. The study was conducted in accordance with the declaration of Helsinki. The study was approved by the institutional review board of Tanta University (IRB approval number: 34296/11/20).

### Eligibility criteria

Patients with lifelong PE with stable monogamous female sexual partners for at least 6 months and declared the ability to follow study instructions and complete study assessment tools were included. Those who suffer from bleeding tendencies, hypersensitivity to botulinum-A toxin or muscular weakness as myasthenia gravis; or suffer from PE secondary to erectile dysfunction, genital infection or psychic stress were excluded from the study. It is to be noted that erectile dysfunction was excluded using the International Index of Erectile Function-5 (IIEF-5) [8, 9].

### Measurements

All patients were evaluated by medical history (age, medical co-morbidities, onset of the condition, duration, previous medications), physical examination to exclude any anomalies in the external genitalia. Also, they were objectively evaluated using intravaginal ejaculatory latency time (IELT), the Premature Ejaculation Profile (PEP) score and female partner’s satisfaction score.

The PEP score includes 4 individual measures: self-perceived control over ejaculation, personal distress related to ejaculation, contentment with sexual intercourse, and interpersonal challenges associated with ejaculation. Each of these measures is evaluated using a five-point response scale. This instrument finds utility in appraising various domains of PE as well as its treatment efficacy [10].

Regarding the IELT, the partner was requested to run a calibrated stopwatch on vaginal penetration and stop it on extra or intravaginal ejaculation or withdrawal of the penis without ejaculation at the end of sexual intercourse. The IELT considered for statistical analysis was the geometric mean of the last 3 sexual acts’ IELT at each time point i.e., pre-injection and 1, 3 and 6 months after injection. It is to be mentioned that all patients received training on how to record IELT using calibrated stopwatch prior to enrollment in the study. Regarding female partner’s satisfaction, it was assessed using a 1 to 5 Likert scale where 1 is very dissatisfied, 2 is dissatisfied, 3 is neither satisfied nor dissatisfied, 4 is satisfied and 5 is very satisfied.

### Randomization

Independent randomization (in 1:1 ratio) was conducted by a third party (not involved in the study) using a computer-generated random table with stratification according to botulinum-A or placebo. The treating physicians were aware of the randomization, whilst patients were unaware.

### Intervention

**Fig. 1:**
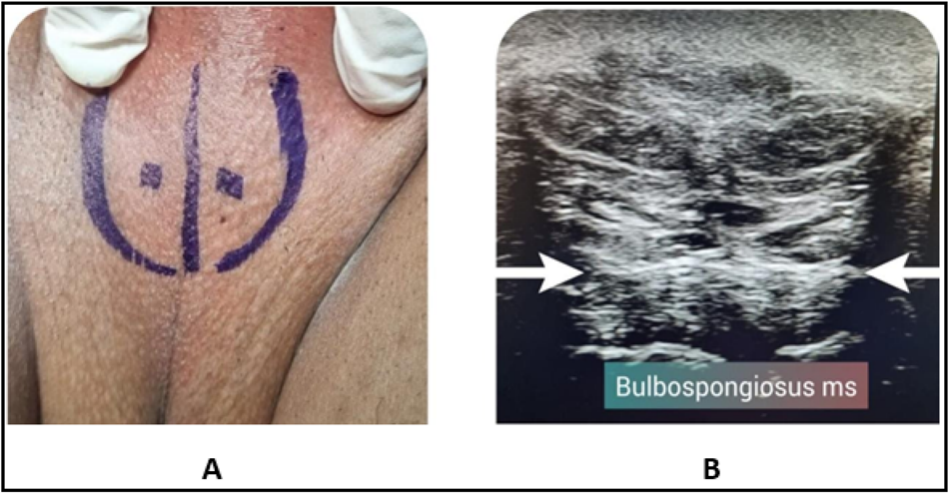
Site of injection. **A** Pre-injection skin marks, **B** US image of bulbospongiosus muscle identification during drug injection.

Eligible patients have been asked to stop any medical treatment that could affect their sexual function e.g., phosphodiesterase 5 inhibitors and medications for lifelong PE; for at least 1 month before injection as well as 6 months thereafter. BS injection with 100 U of botulinum-A toxin in 10 ml of saline was performed in group I (treatment) whereas Group II (placebo) was injected with 10 ml of saline in BS. Injection was done in lithotomy position, under ultrasound guidance using the superficial probe to localize the site of injection; by experienced uro-andrologist. No anesthesia was required as the injection was well tolerated by all patients. Injection was done under complete aseptic condition, in 2 points, one on each side of the midline in order to infiltrate the right and left muscles. The drug was distributed all through the muscle using fanning technique and ultrasound guidance ensured accuracy of infiltration (Fig. 1A, B).

### Measured outcome

The primary outcome was to assess the effect of the injection of botulinum-A toxin in BS on the PE status. This was performed by reporting changes in IELT, PEP and female partners’ satisfaction before treatment and at 1, 3 and 6 months thereafter. We compared the changes in the mean values between both groups at the determined follow-up points. The second outcome was to assess the safety of the drug injection by reporting any adverse events during or after injection in both groups.

### Statistical analysis

Pre allocation sample size estimation was not performed because of unavailability of previous similar studies and the expected limited number of eligible patients. Post-hoc sample size assessment was performed considering the mean difference in PEP score changes between both groups at 1 month (effect size: 0.6) and alpha error 0.05. The estimated power was 80%. The per-protocol analyses were performed to avoid the impact of dropped-out cases. Independent sample t test was used to compare the mean scores between both groups at different intervals. The changes in the mean PEP, IELT and female satisfaction scores at different time points in each group were done using paired sample t test. The comparison of the changes in the mean scores in relation to baseline value between both groups was done by repeated measure ANOVA test. Statistical analysis was performed using IBM SPSS software v. 21. A *P* value of <0.05 was taken to indicate statistical significance.

## Results

**Fig. 2:**
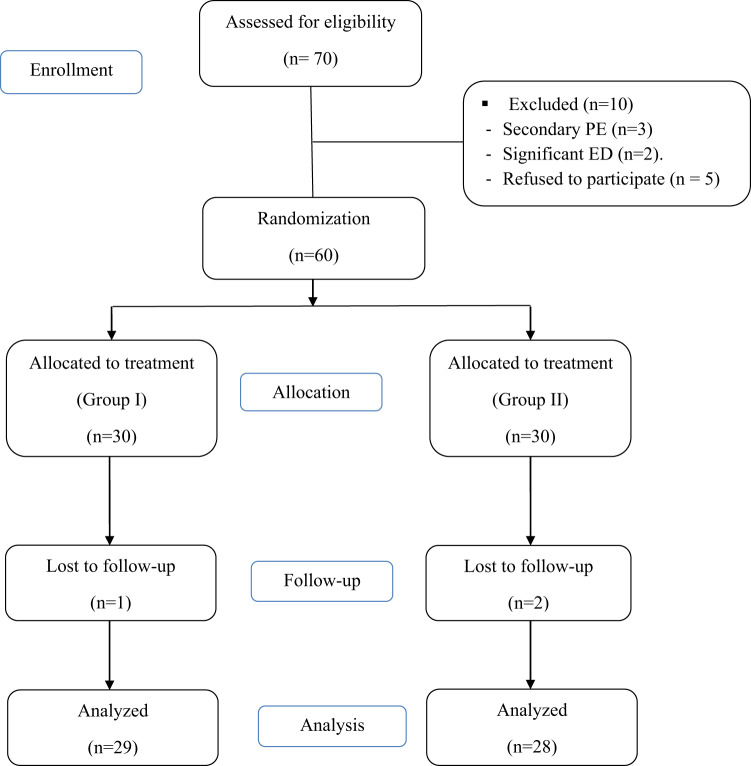
CONSORT Flow chart of the study population.

A total of 60 patients have been enrolled in the study: 30 in each group. However, 57 completed the follow-up protocol and the other 3 were lost in follow-up. Fig. 2 shows the flowchart of the study population. At baseline, there were no statistically significant differences between both groups in terms of patients’ and partners’ age, baseline PEP (*P* = 0.4), IELT (*P* = 0.6) or partner satisfaction (*P* = 0.5) (Table 1).

**Table 1:** The demographic criteria and outcome measures in botulinum A-toxin and control groups.

Mean ± SD	Botulinum-A toxin (Group I) *N* = 29	Placebo (Group II) *N* = 28	*P* value
Age. *Years*	38.7 ± 7.3	39 ± 9	0.6
Female age. *years*	33.6 ± 7	35.5 ± 9.6	0.4
Co-morbidities^a^			0.9
DM	1 (3.3%)	1 (3.3%)	
HTN	2 (6.7%)	1 (3.3%)	
IHD	1 (3.3%)	1 (3.3%)	
IIEF-5	22.4 ± 1	22 ± 1.5	0.5
Baseline PEP	2.3 ± 0.8	2.5 ± 0.9	0.4
1-month PEP	3.1 ± 0.7	3 ± 1.1	0.9
3-month PEP	2.9 ± 0.3	2.8 ± 0.8	0.9
6-month PEP	2.4 ± 0.6	2.6 ± 0.1	0.6
Baseline IELT	31.4 ± 9	34.2 ± 13	0.6
1-month IELT	33.1 ± 11	35.6 ± 7	0.6
3-month IELT	33 ± 7	35.4 ± 8	0.5
6-month IELT	31.9 ± 10	34.8 ± 12	0.5
Baseline female satisfaction	2 ± 0.8	2.1 ± 0.8	0.5
1-month female satisfaction	2.2 ± 0.9	2.3 ± 0.9	0.6
3-month female satisfaction	2.1 ± 0.9	2 ± 0.1	0.8
6-month female satisfaction	2 ± 0.4	2.2 ± 0.7	0.3

All variables were expressed as mean ± SD and comparisons were performed using independent sample t test.

^a^The comparison was done by Chi-square test. *DM* diabetes mellitus, *HTN* hypertension, *IHD* ischemic heart disease, *IIEF-5* International index of erectile function-5, *IELT* intravaginal ejaculation latency time, *PEP* premature ejaculation profile

In group I, the mean of PEP increased significantly at 1- and 3- month of follow-up (*P* = 0.02 and 0.04, respectively) but insignificant increase was noted at 6-month (*P* = 0.6). However, at 1-, 3-and 6-month of follow-up; there was no statistically significant change in the IELT (P = 0.2, 0.2 and 0.5, respectively) or female partner’s satisfaction (*p* = 0.5, 0.9 and 0.8, respectively).

In group II, there were insignificant changes in the mean PEP at 1- (*P* = 0.06), 3-(*P* = 0.1) and 6-month (P = 0.6). Similarly, insignificant changes had been noted in the IELT (*P* = 0.06, 0.09 and 0.3) and female partner’s satisfaction (*P* = 0.08, 0.8 and 0.1) throughout the study duration after 1, 3 and 6 months respectively.

**Fig. 3:**
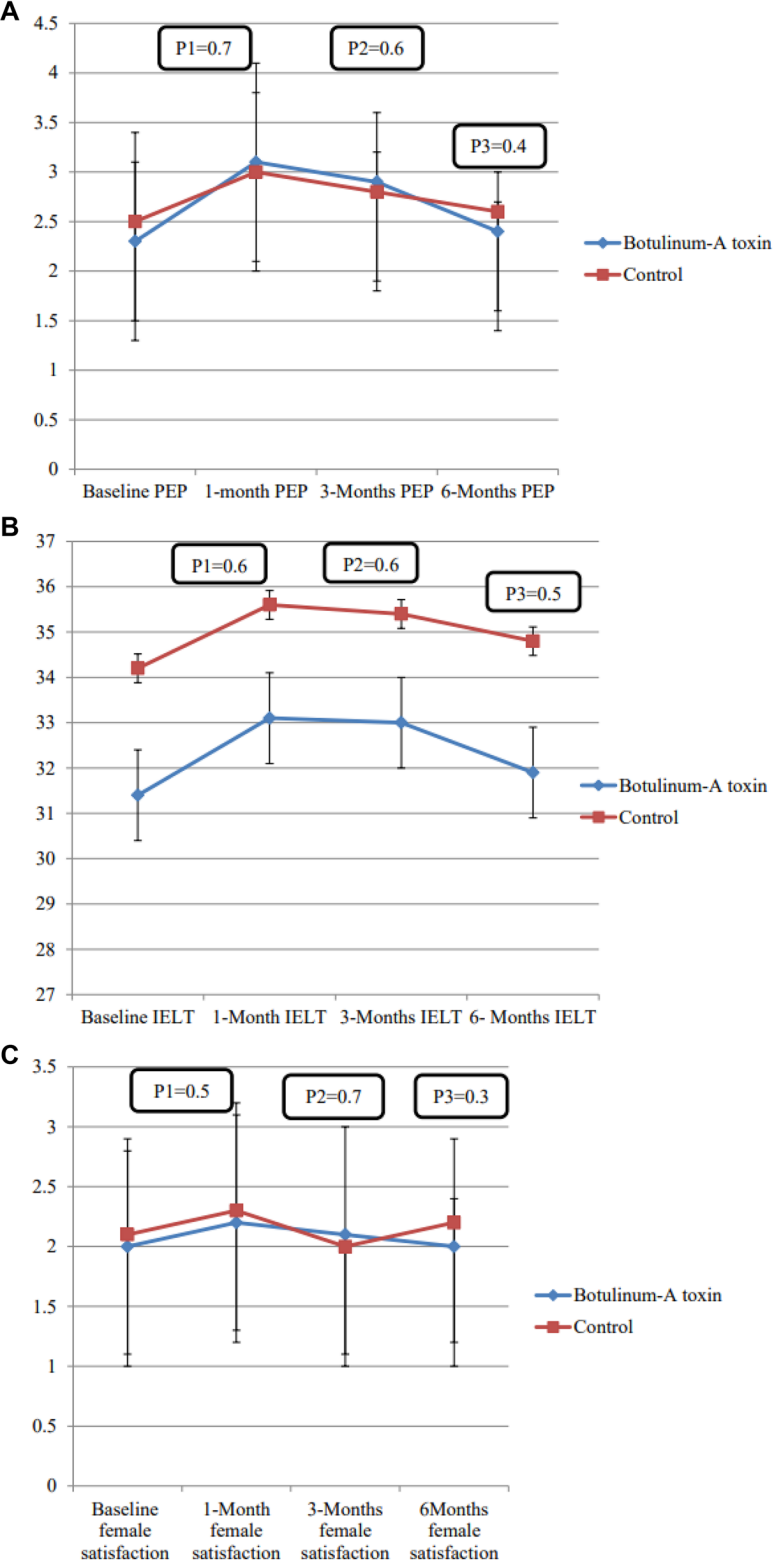
Efficacy outcome. Changes of PEP (**A**), IELT (**B**) and female satisfaction (**C**) in both groups. P1 illustrates the differences in mean changes between baseline and 1-month, P2 illustrates the differences in mean changes between baseline and 3- months and P3 illustrates the differences in mean changes between baseline and 6- months values (Repeated measure ANNOVA test).

When comparing both groups, there were insignificant differences in the changes of the mean PEP score at 1-,3- and 6-months (*P* = 0.7, 0.6 and 0.4, respectively) (Fig. 3A). Also, no statistically significant differences were found regarding the changes in the mean IELT (*P* = 0.6, 0.6 and 0.5) (Fig. 3B) and female partner’s satisfaction score (*P* = 0.5, 0.7 and 0.3) throughout the study period (Fig. 3C).

Adverse events were observed only in 3 cases (5.3%); 2 in group I and a single case in group II (*P* = 0.5). One patient in group I suffered from mild erectile dysfunction which lasted for 1 month, where there was a reduction in his rigidity, but he was still able to have penetrative sex. Another patient in the same group reported post-micturition dribbling which occurred from day 4 after injection and lasted for 2 months. A patient in the control group developed mild urethral bleeding post-injection which stopped spontaneously after 2 days.

## Discussion

In 2010, the theory of using botulinum-A toxin for treatment of PE was postulated [7]. It was thought that injection of botulinum-A toxin in BS will inhibit rhythmic contractions and may prolong ejaculatory latency. Theoretically, the expulsion phase only of ejaculation is affected by injection of botulinum-A toxin into BS, while the emission phase should stay unaffected [7]. In 2014, Serefoglu et al. injected botulinum-A toxin percutaneously into the BS of male rats. They reported an increase in ejaculatory latency times, but the difference in post-treatment ejaculatory latency between botulinum-A toxin injection and sham groups was not statistically significant [11]. In a pursuit to explore the effect of botulinum-A toxin on the emission and expulsion phases of ejaculation in rats, Ongün et al. revealed a remarkable revelation. Rats graced with the administration of 5 units of botulinum-A toxin showed significant delay in their ejaculation compared to sham group (mean ± SD = 1092 ± 657 seconds, versus 298 ± 81 seconds, respectively) [12].

The only clinical trial that assessed BS injection of botulinum-A toxin effect for the treatment of lifelong PE in humans was done by Li et al. in 2018. They randomly assigned 70 patients with lifelong PE to a treated (100 U botulinum-A was injected) and a control group (saline was injected). The study group showed a significantly longer IELT at 4 weeks after treatment compared to the control group, and also had significant improvement in PEP-ejaculation scores, PEP-sexual satisfaction, PEP-PE-related distress, and PEP-PE-induced difficult relationship with the partners. The female partners of the treated group also showed significant improvement in sexual satisfaction scores [13].

Herein, we identified a significant improvement in the PEP score in the treatment group compared to the baseline. The improvement in the PEP score was not associated with a statistically significant improvement in the IELT or female partner’s satisfaction. However, we could not identify a significant difference between the botulinum-A toxin-treated group and the placebo group in any of the tested scores. To be noted that none of our patients accepted any recommendation of reinjection.

Li et al. observed adverse effects in 6 cases (17.6%) including 4 cases of decreased erectile hardness (11.76%) and 2 of incomplete urination (5.88%) [13]. Adverse effects in the current series were reported only in 3 cases (5.3%) of the study population; 2 patients in the treatment group suffered from loss of rigidity during erection and post-micturition dribbling and one patient in the placebo group had mild bleeding per urethra which stopped spontaneously. Of note, all patients reported normal ejaculation; no dribbling of ejaculate was encountered in any of them.

To the best of our knowledge, this is the second study in the literature investigating the effect of botulinum-A toxin injection into BS for treatment of lifelong PE and the first to deny its clinical efficacy. However, it has some limitations. We used the non-validated Arabic version of PEP questionnaire because of unavailability of Arabic validated form. Another important point is that we chose to inject the drug into the BS muscle in a single site on both sides and infiltrate the drug in a fanning technique under ultrasound guidance. This may explain the differences in our results compared to Li et al. [13]. Whether or not injection into multiple points would change the outcome is a point that should be investigated in further studies.

## Conclusions

Injection of botulinum-A toxin into the BS muscle seems to be safe but failed to prove clinical efficacy for treatment of lifelong PE when compared to placebo. However, further studies with larger sample size may be needed to validate this conclusion and find other novel lines of treatment.

## Data Availability

The datasets generated during and/or analyzed during the current study are available from the corresponding author upon reasonable request.
